# Using a neural network model to evaluate the mechanical and tribological properties of vermicular cast iron based on hardness

**DOI:** 10.1016/j.heliyon.2023.e21119

**Published:** 2023-10-18

**Authors:** Gamal M.A. Mahran, Abdel-Nasser Mohamed Omran

**Affiliations:** a Mining Engineering Department, Faculty of Engineering, King Abdulaziz University, Jeddah, 21589, Saudi Arabia; b Mining and Petroleum Department, Faculty of Engineering, Al-Azhar University, Qena, 83513, Egypt

**Keywords:** Neural network, Cast iron, Wear, Impact, Hardness

## Abstract

Measuring the tensile strength, wear resistance, and impact strength of metals, particularly cast iron, is complex and more expensive than performing hardness tests. In the present study, owing to the ease of specimen preparation and low cost, the Hardness (HB) test was used to approximately predict Wear Rate (WR), Impact Energy (IE), and tensile strength (TS). The relation between Mg% and HB, tensile strength, WR, and IE was examined by using three experimental groups of compacted graphite cast iron (CGI) treated with a nodulizer (Fe–Si–Mg) alloy at different carbon equivalents (CEs) of 3.5, 4.0, and 4.5 %. The produced CGI exhibited HB, TS, WR, and IE of 191–226 HB, 402–455 MPa, 30.1–23.8 mg/cm^2^, and 22–15 J, respectively. The good results were taken at a CE of 4.5 % and Mg content of 0.0118–0.0155 %. the regression analysis and artificial neural network model (ANNs) were used in the hardness test, and the results indicated the possibility of predicting IE, WR, tensile strength, and high accuracy Mg% of the produced CGI. It could be observed that, the neural network algorithm model has a high prediction precision for determining the Mg% content and the properties of the prepared CGI based on hardness. In the case of CE = 4, the MSE calculated for the predicted and measured data taken from the used ANNs model is 3.7 E−8, 20.33, 0.3084, and 0.099 for Mg%, TS, WR, and IE, respectively.

## 1 Introduction

Compacted graphite iron (CGI) belongs to an advanced cast iron group characterised by short-sized graphite with round edges within the iron matrix. It exhibits good mechanical, tribological, and physical properties and is an intermediate between grey iron or flake graphite iron (FG) and spherical graphite iron (SG) [1,2]. According to the literature, CGI exhibits a minimum improvement of 60–75 % in tensile strength and 35 % in elastic modulus and approximately twice the fatigue and impact strength of common FG. CGI also outperforms SG in terms of some physical properties, including thermal conductivity and damping capacity [3,4]. CGI is acceptable if FG is not included in the structure, the vermicular graphite volume is greater than 80 %, and the spherical graphite volume is less than 20 % [5,6]. CGI is easier to cast than SG and exhibits a higher thermal conductivity and damping capacity [6,7]. he high thermal resistance of shock of CGI encourages it to be suitable for many applications, such as brake components and internal combustion engines [[8], [9], [10]]. Because CGI is more resistant to wear than FG, it is used for brake components with a high friction coefficient [11,12]. Many factors influence CGI production, including casting time, temperature, casting thickness, magnesium content (Mg%), and carbon equivalent (CE). The percentage of Mg is the most important factor. When the Mg content in the cast is less than 0.008 %, FG is formed, and when it is more than 0.02 %, SG is formed [11,[13], [14], [15], [16]]. However, when the Mg content is between these, CGI with varying nodularity percentages is formed [6,17,18]. CGI has rekindled interest in foundries owing to its useful mechanical and physical properties and resistance to thermal shock. Therefore, it is used to develop engine blocks for internal combustion engines [8,19]. Artificial neural networks (ANNs) have applications in many disciplines, including prediction through practical experiments in the form of input [20,21]. Artificial neural network modeling is used in many applications. One of the authors mentioned that it is used to predict silicon as an alloying agent in the process of producing cast iron in furnaces [22]. Other authors have reported that the properties of CGI can be predicted by their relationship to chemical composition and microstructure [23]. The human brain's network inspired the concept of an ANNs, which is composed of many layers and input layers, with the most common being a single layer and intermediate layers of physical quantities known as hidden layers [19,24]. The hidden layer consists of one or multi layers of neurones, and the output layer is the final layer that produces the expected values. The hidden layers are treated by error calculating the sum of the input and output. The hidden layers use the weighted sum of the input layers to activate or train the proposed neural network, which considers the inputs and their weights for each input in each neuron. Neurones generate outputs that are fed into the next layer, a process known as forward propagation. An ANNs activation or training aids neurones in learning data patterns [25,26]. The outputs of the input layer are passed to the next hidden layer, and this is repeated to obtain products from the output layer. Network parameters are often developed to minimize the sum of squared errors of approximation [27,28]. The advantages of ANNs include the ability to draw linear and polynomial relationships and predict these relationships directly from the inputs used. Another advantage of ANNs is the ability to model complex relationships without the use of simplistic hypotheses, as is common in linear methods [26,27,29]. When compared to other tests, such as tensile, wear, and impact testing, which require high-cost accurate machining, the hardness test is among the best and easiest mechanical tests in terms of cost and preparation because it only requires a flat and polished surface [[30], [31], [32]].

In this work, three batches of CGI were fabricated in this study by varying the CE with different Mg contents. The required mechanical characteristics, such as TS, HB, WR, and IE were examined. The present work aims to predict the correlations between hardness and TS, WR, and IE results of these groups using regression analysis and the ANNs model. Then the comparison between the predicted and actual values of TS, WR, Charpy IE results, and Mg% content was estimated as a function of hardness.

## 2 Experimental work

### 2.1 Materials

Steel scrap, carbon, pig iron, ferrosilicon, and nodulisers were used in this study. The elemental analyses of these materials are presented in Table 1.

**Table 1:** Elemental analysis of the utilized materials.

Item	C%	Si%	Mg%	Mn%	Cu%	Fe%
Pig iron	4.05	0.2	0.0001	0.12	0.002	Bal.
Steel scrap	0.16	0.35	0.0001	0.11	0.0001	Bal.
Carbon	89.1	–	–	–	–	–
Fe–Si	0.5	75	–	0.2	–	Bal.
Copper	99.99	–	–	–	–	–
Noduliser	0.2	46	5.9	–	–	Bal.
Fe–Mn	–	–		75	–	Bal.

### 2.2 Experimental procedure

**Fig. 1:**
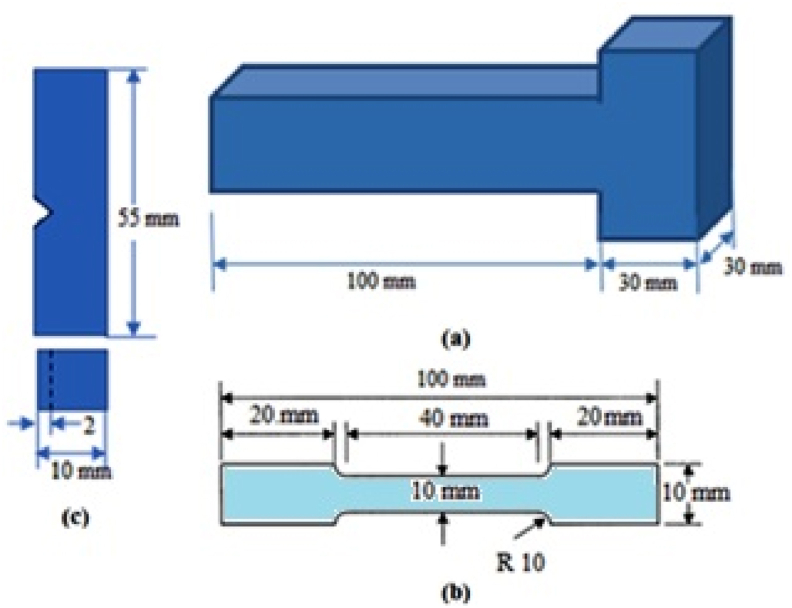
Schematic diagram of a) Mould dimension, b) Tensile test specimen, and c) Charpy impact specimen.

Steel scrap and pig iron in the amount of 3 kg were shipped into the crucible of a high-frequency furnace even the mixture was melted completely. the melt was treated using added a flux to the melt and the formed slag was removed. The molten cast iron was maintained at the needed temperature (1400 °C), and the melt was analysed using a CE metre to account for the C and Si% in the melt. To prevent magnesium oxidation, the noduliser (Fe–Si–Mg) was encapsulated by steel foil (steel drink can) and injected into the crucible containing the molten metal following the primary alloying elements were completely adjusted. The molten metal was then poured into a pre-prepared sand mould at approximately 1350 °C, as shown in Fig. 1a. Cast thickness 30 mm, casting time 2 min, casting temperature 1350 °C at different CEs (3.5, 4.0, and 4.5), and Mg content was changed from 0.0066 to 0.022 % Mg were all kept constant. To obtain different mechanical and tribological properties, the casts were cut and machined in accordance with ASTM standards. The tensile test was performed on a SHIMADZU (VH–F1000 KN) uniaxial testing machine, and the specimens were cut and machined in accordance with ASTM standards, as shown in Fig. 1b, the wear-weight loss of the CGI was measured using a pin-on-disk testing device. The used discs were made of grey cast iron, and the loss of specimen weight was collected, weighed, and calculated in relation to the sliding time and weight loss at a constant load of 4.8 Kgf, at the same sliding speed of (2.5 m/s) for 5 h. The wear test pins were rod-like pins 25 mm long and 5-mm in diameter on a flat disc made of common grey cast iron. As shown in Fig. 1c, Charpy impact test specimens were prepared according to the ASTM standard size (10 × 10 × 55 mm).

#### 2.2.1 ANNs model

**Fig. 2:**
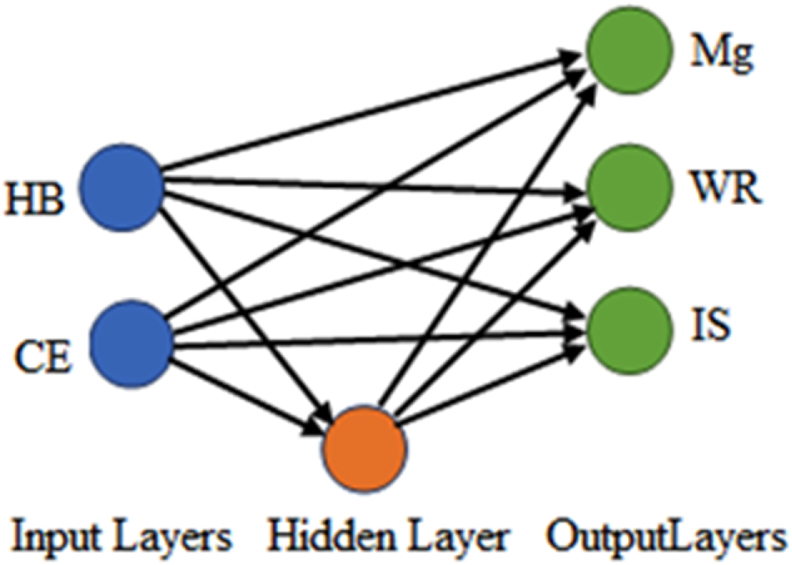
Cascade-forward-back-propagation ANNs model that is trainable to predict the material properties of the produced CGI based on the hardness and CE.

The addition of a nodulizer to cast iron as an Mg source resulted in the production of CGI. Mechanical and tribological properties were determined by performing measurements. The percentage of Mg in the produced cast iron was used as an independent variable, and it was correlated to previously mentioned cast iron properties such as HB, TS, IE, and WR. Fig. 2 shows the CFBP system's diagram, which is used to predict the percentage of Mg, TS, impact strength, and WR in the prepared CGI based on its hardness. The Mg% in CGI, for example, is assumed to be a function of hardness, TS, IE, and WR. The neurons' number in the output and input layers is equal to the neurons' number in the output and input layers of the hidden layer Thus, the number of neurons in Mg% equals one, and in the hidden layer, the number of neurons has a significant effect on the ANNs's accuracy. Only one hidden layer is considered in the proposed model, which approximates any type of nonlinear relation and appropriate transfer functions. Moreover, the sigmoid log function was used in the hidden layer, and the pure linear transfer function (PureLine), was used in the output layer. This model was then trained to correct errors and predict the TS, WR, IE and magnesium content of the machined CGI using hardness. Also in this study, a trainable cascade back propagation network was used.

## 3 Results and discussion

### 3.1 Hardness, tensile strength, wear rate, and impact energy results

**Fig. 3:**
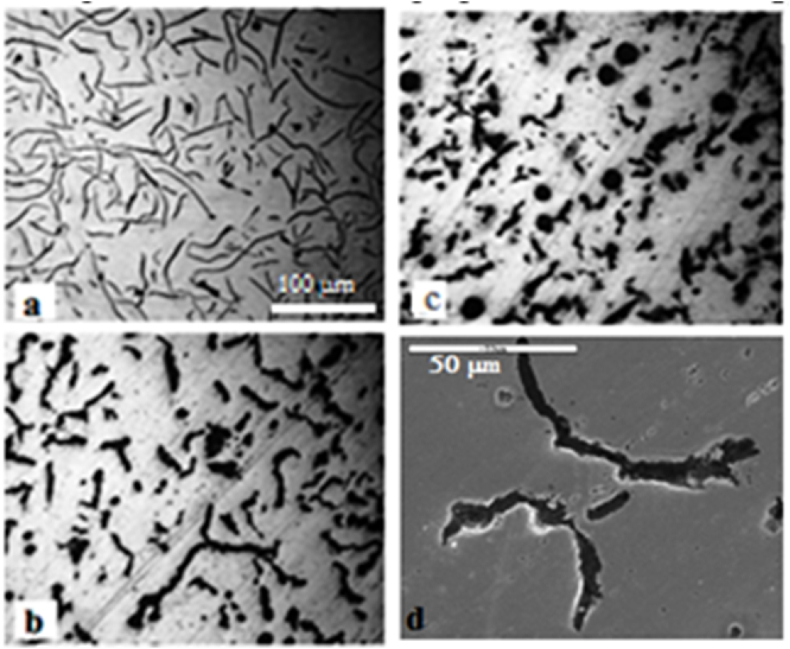
LOM micrographs of the produced cast iron with a) 0.0066 % Mg, b) 0.0118 % Mg, and c) 0.0155 Mg and d) SEM micrograph of 0.0118 % Mg.
b) According to the literature, as the Mg content wt% increases (from 0.0058 to 0.025 % in the prepared CGI, the carbon shape changes from a flaky shape to a vermicular shape and then to a spheroidal shape, the HB and TS values increase, whereas the IE (toughness) and WR values decrease. In comparison to the untreated FG, the Mg treatments produced good results. The change in carbon shape from flack-like to vermicular with round ends or a nodular shape resulted in a uniform distribution of stress and improved mechanical properties, as shown in Fig. 3a, b, c, and d.

**Fig. 4:**
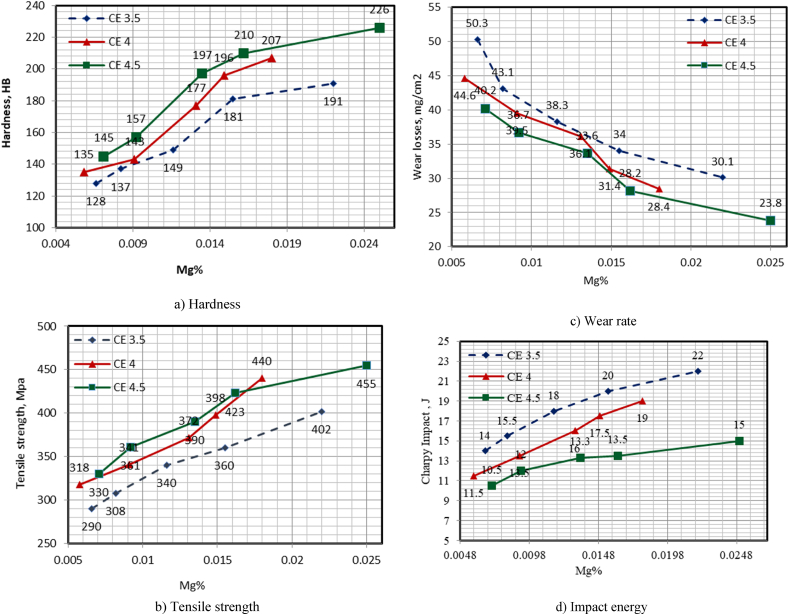
(a–d) Effect of Mg contents on the mechanical properties of the prepared CGI in various case studies.

Brinell hardness (HB), TS (MPa), WR (mg/cm^2^), and IE (J) of CGI produced with different nodulizer content and different CEs of 3.4, 4.0, and 4.5 %. The following are some of the findings:

a) Fig. 3a–d: Light optical microscopy (LOM) images of cast iron with varying Mg contents. The photography (Fig. 3 a) exhibits a Mg content of 0.0066 %, and the carbon shape is a flack-like that of carbon particles inside the cast iron matrix is known as flake graphite (FG) iron; when the magnesium content increases to 0.0118 % Mg (Fig. 3b), the flak-like carbon is converted to a vermicular shape, which is known as CGI. Fig. 3c shows a micrograph of a cast iron specimen with Mg content of 0.0155 %, it is clear that some of the spherical carbon particles exhibited 15 % nodularity. As the Mg content increased, so did the nodularity, which may lead to an increase in the mechanical characteristics and a decrease in the physical characteristics and toughness [33]. Fig. 3d depicts a scanning electron microscopy (SEM) image containing 0.0118 % Mg. According to Kim et al. the carbon particles, like a worm with a round edge, caused a uniform distribution of stress, which increased the mechanical properties [33].

c) The HB values were 128–145, 149–197, and 191–226 HB for FG, CGI, and SG, respectively. Increasing the magnesium content of the produced cast iron from 0.0058 to 0.025 % improved the HB with different CEs of 3.5, 4.0, and 4.5 by 49.22, 53.33, and 55.86 %, respectively (Fig. 4a). This could be due to the presence of Mg, which converts flake graphite to vermicular graphite, and a higher Mg content increases nodular graphite (SG) [1,5]. d) TS values were 290–330, 340–390, and 402–455 MPa for FG, CGI, and SG, respectively. Increasing the magnesium content in the produced cast iron from 0.0058 to 0.025 % improved the TS with varying CEs of 3.5, 4.0, and 4.5 by 38.62, 38.36, and 37.88 %, respectively (Fig. 4b). This could be because the higher Mg content increases modularity [33]. e) The WR values were 50.3–40.2, 37.3–33.6, and 30.1–23.8 mg/cm^2^ for FG, CGI, and SG, respectively. Moreover, increasing the Mg% in the produced cast iron from 0.0058 to 0.025 % significantly reduced the WR with CEs of 3.5, 4.0, and 4.5 by 40.16, 36.32, and 40.8 %, respectively (Fig. 4c). This could be due to the same reason as previously stated. f) The IE values were 10.5–14, 18–13.3, and 22–15 J for FG, CGI, and SG, respectively. Furthermore, increasing the Mg content in the produced cast iron from 0.0058 to 0.025 % improved the IE with different CEs of 3.5, 4.0, and 4.5 by 57.14, 65.12, and 42.86 %, respectively (Fig. 4d). This could be due to the transition from FG to CGI and the increase in modularity. Although the IE is inversely proportional to the increase in CE, it increases with the change in carbon shape from flak to vermicular due to increased ductility as nodularity increases.

### 3.2 Regression analysis of TS, WR, and IE results with HB

The following is the relations between TS (Mpa), WR (mg/cm^2^), and IE (J) with HB values for all experimental results and for each case of CE for CGI:

#### 3.2.1 Correlation of TS with hardness values for all experiments and each case of CE

**Fig. 5:**
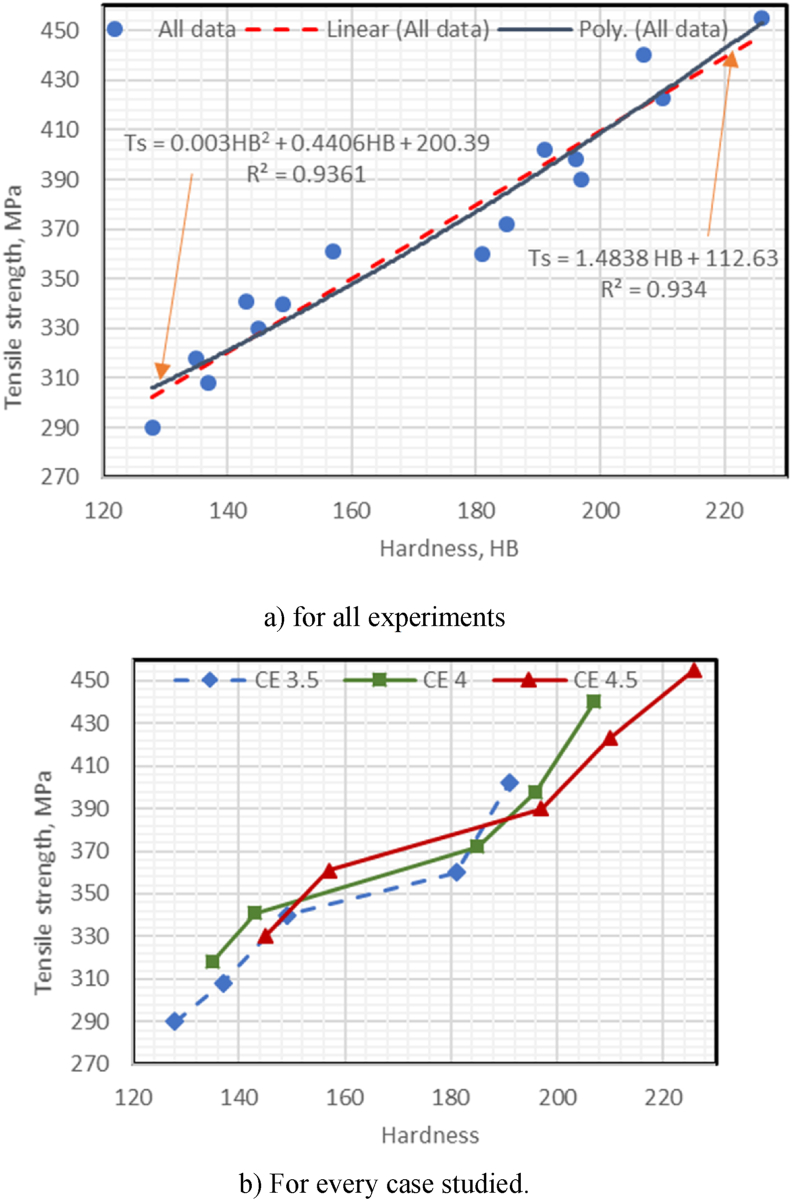
(a–b): Relation between Tensile strength with hardness.

According to Fig. 5 (a), the TS appears to be highly linearly correlated with HB, with TS = 1.484 HB + 112.63, R^2^ = 0.934, and standard error = 13.06 MPa. If the correlated trend alters from linear to polynomial, then TS = 0.003 HB^2^ + 0.4406 HB + 200.39, with R^2^ = 0.936. This coefficient is nearly identical and provides a good fit.

- The polynomial trend between TS, Brinell hardness, and HB of CGI produced at different CE values of 3.5, 4.0, and 4.5 produced good results in each case of CE. Fig. 5b indicates that increasing CE improved the TS of CGI (i.e., R^2^ = 0.963) at CE = 4.0, compared to CGI at CE = 3.5 (i.e., R^2^ = 0.929) and a greater increase in CE to 4.5 yields the best result (R^2^ = 0.967).

#### 3.2.2 The correlation of WR with HB values for all experiments & each separate case of CE

**Fig. 6:**
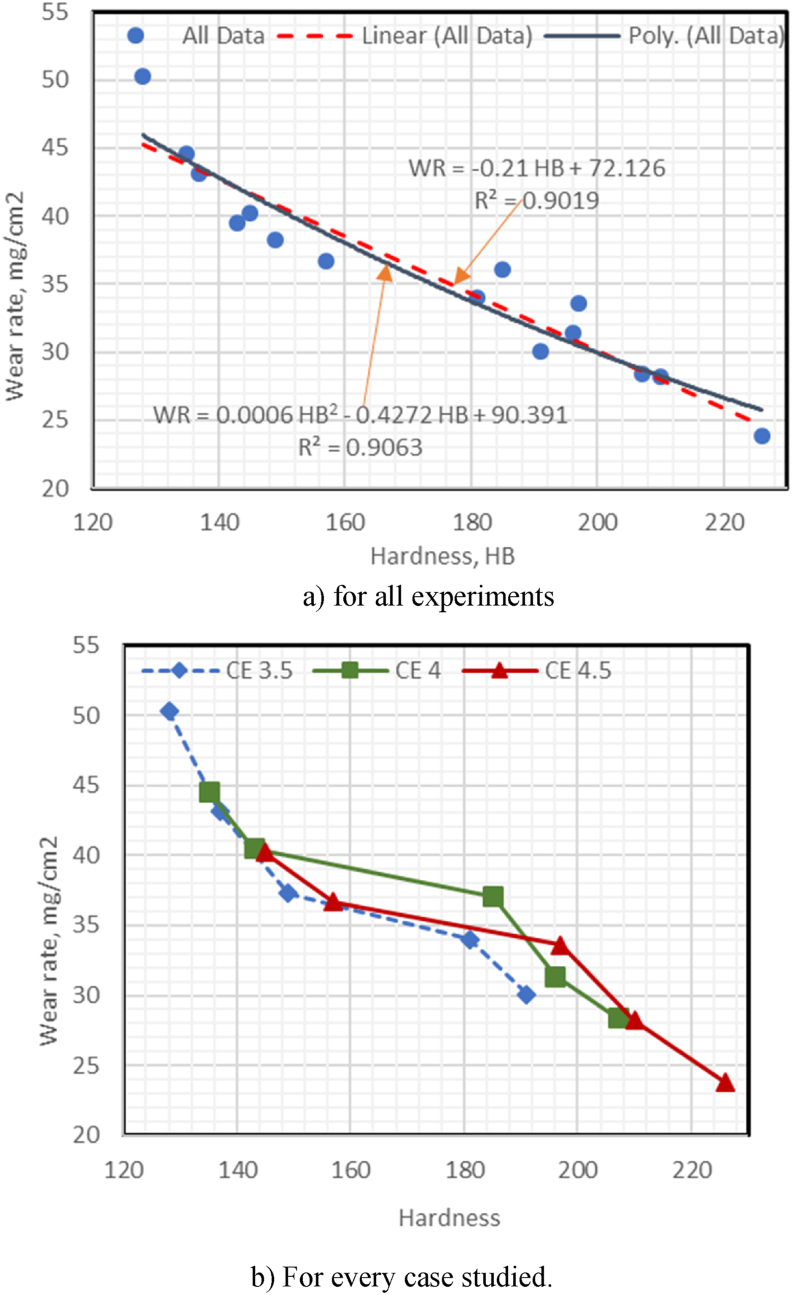
(a–b): Relationship between wear rate and hardness.

Fig. 6a shows that the WR have a good linear correlation with HB, as WR = −0.21 HB + 72.126 with R^2^ = 0.9019 and a standard error of 2.29 mg/cm^2^. WR = 0.0006 HB^2^ − 0.4272 HB + 90.391, with an R^2^ of 0.9063 if the linear correlation is changed to a polynomial trend. When comparing polynomial trend correlation to linear correlation, there is a slight improvement, but the results obtained from all data for CGI experiments in both linear and polynomial relationships a good fit is not given [34]. As shown in Fig. 6b, the relationships of the polynomial trend between the WR, and HB of the CGI obtained at different CE values of 3.5, 4.0, and 4.5 produced exceptional results for each case of CE. Increased CEs (3.5, 4.0, and 4.5 %) improved the CGI WR (i.e., R^2^ = 0.952, 0.941, and 0.962, respectively).

**Fig. 7:**
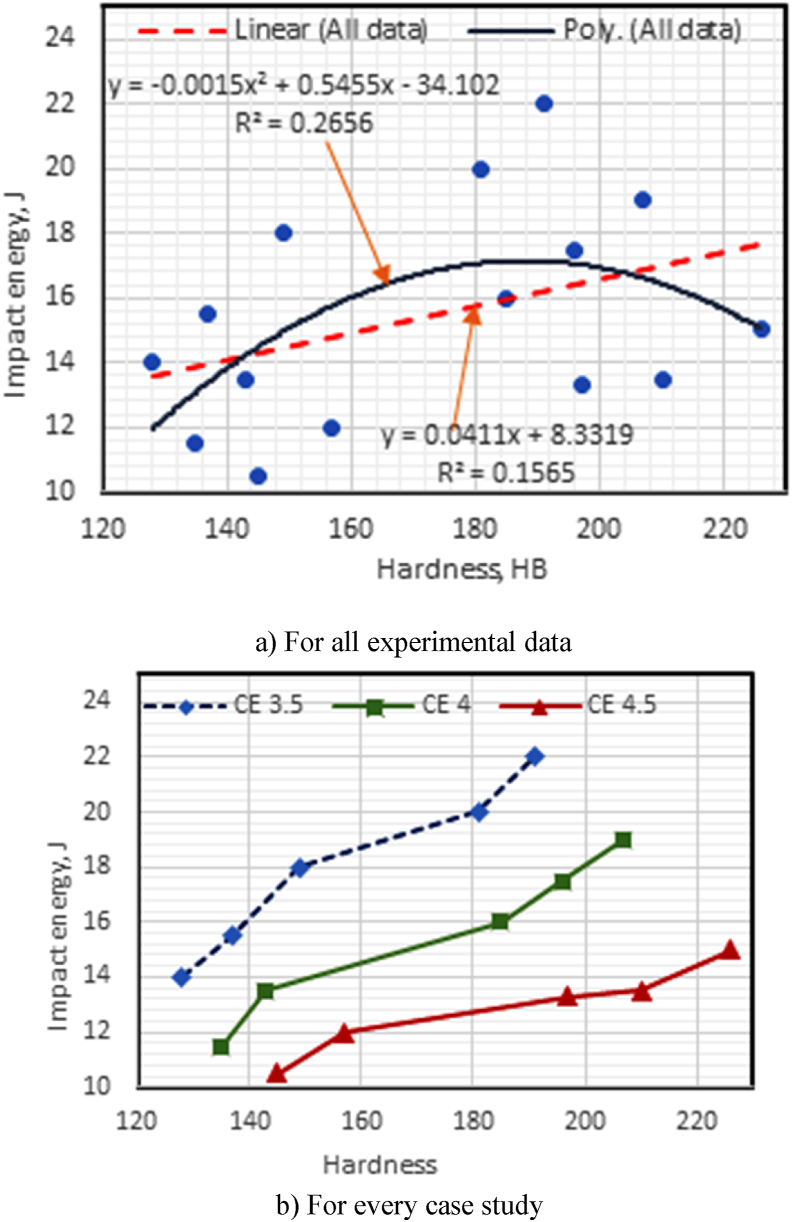
(a–b): Correlation between impact energy with hardness in the prepared CGI.

#### 3.2.3 Correlation of impact energy with hardness values for all experiments and each case of CE

- Fig. 7a shows that the IE has a poor linear correlation with HB, with IE = 0.0411HB + 8.3319, R^2^ = 0.1565, and the standard error = 3.16 J if the linear correlation changed to a polynomial trend, then IE = - 0.0015 HB^2^ + 0.5455 HB - 34.102 and R^2^ of 0. 266. When comparing linear trend correlation with polynomial correlation, there is a slight improvement, but the obtained results for all data of the CGI experiments in linear and polynomial relations a good fit is not obtained. The smaller coefficient R^2^ values could be attributed to the use of all data at different CE and cast-iron types.
- As the CE in the prepared CGI increases (3.5, 4.0, and 4.5), the impacts energy of CGI decreases; therefore, R^2^ decreases where R^2^ (0.969, 0.966, and 0.936, respectively). The higher the coefficient of R^2^ in these findings, the better the results (Fig. 7b).

Thus, in all combination cases studied, hardness is the independent variable, whereas the other properties, TS, WR, and IE, are dependent variables affected by the amount of nodulizer and the CE used. R^2^ values of for all combination cases studied show a strongly positive relationship, with increasing slope indicating significant and strong relationships between the three variables (TS, IE, and WR and HB) as indicated in Table 2.

**Table 2:** Tensile strength, Impact strength, and Wear rate datasets versus hardness for CGI using regression analysis.

Property Cases studied	Tensile strength		Wear rate (WR)		Impact energy (IE)	
	Good fitting equation	R^2^	Good fitting equation	R^2^	Good fitting equation	R^2^
overall data	TS = 0.003 HB^2^ + 0.4406 HB + 200.39	0.936	WR = 0.0006 HB^2^ – 0.4053 HB + 88.572	0.894	IE = −0.002 HB^2^ + 0.5455 HB –34.102	0.266
CE 3.5	TS = −0.0033 HB^2^ + 2.6014 HB + 14.883	0.9285	WR = 0.0049 HB^2^–1.8441 HB + 204.86	0.959	IE = −0.001 HB^2^ + 0.423 HB – 24.314	0.9689
CE 4.0	TS = 0.0245 HB^2^–6.905 HB + 813.18	0.9629	WR = −0.0015HB^2^ + 0.3162HB + 27.329	0.928	IE = 0.0004 HB^2^–0.0325 HB + 9.8261	0.9662
CE 4.5	TS = 0.009 HB^2^–1.913 HB + 427.22	0.9671	WR = −0.0019 HB^2^ + 0.5118 HB + 4.5362	0	IE = 3E–05 HB^2^ + 0.0361 HB + 5.0197	0.9357

### 3.3 ANNs model

Table 3 shows a comparing between the predicted Mg% obtained using the constructed ANNs and Mg % obtained from measured data. The MSE is utilized to support the ANNs performance by providing a calculated value coming from the variation between the actual and predicted data extracted from the ANNs. For Mg%, TS, IE, and WR, the MSE at CE = 4.0 is 3.7E–8, 20.33, 0.3084, and 0.099, respectively. The low value of MSE shows that the proposed ANNs model has been successfully applied for the prediction of the outputs. The outcomes exhibit that the proposed ANNs model provided good accuracy in estimated Mg% and IE, but adequate accuracy in terms of wear rate.

**Table 3:** Comparing the measured and computed reading using ANNs for Mg content in the produced CGI, Ts, WR, and IE based on the HB.

Hardness, HB	Mg %		Tensile strength, MPa		Wear rate (WR), mg		Impact energy (IE), J	
	Measured	computed	Measured	computed	Measured	computed	Measured	computed
135	0.0066	0.0065	318	323.44	44.6	44.93	11.5	11.77
143	0.0082	0.0085	341	333.29	39.5	40.12	13.5	13.38
177	0.0116	0.0119	372	375.18	36.1	35.97	16	16.22
196	0.0155	0.0156	398	398.59	31.4	31.40	17.5	17.14
207	0.022	0.0221	440	438.51	2**8**.4	27.39	19	18.52
MSE	0.000000037		20.34		0.308		0.099	

**Fig. 8:**
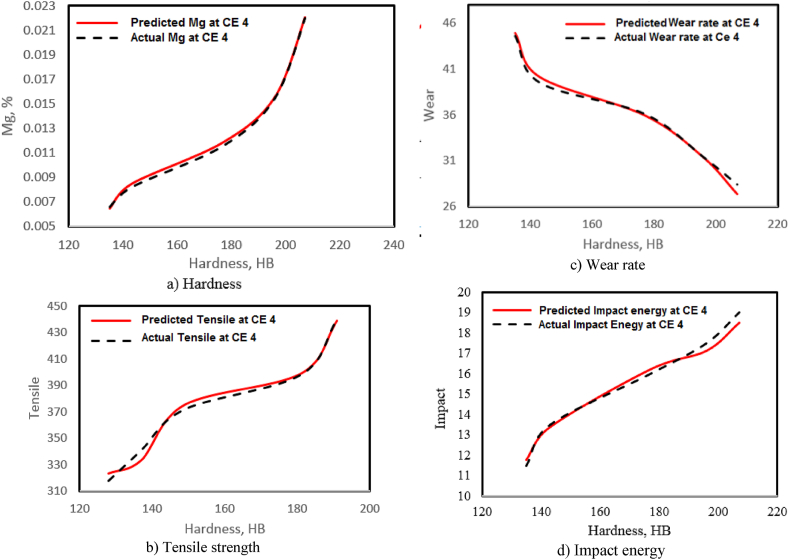
(a–d): Correlation between measured and computed value using ANNs model for Mg% in CGI, tensile strength, wear rate, and impact energy versus hardness at CE 4.0.

Fig. 8(a–d) depicts the correlation between the measured and computed Mg%, TS, WR, and IE of the produced CGI by the suggested ANNs model based on the HB in the case of CE = 4. The standard MSE reveals the good accuracy outcomes for the suggested ANNs model, where the computed MSE for this case (CE = 4) is 3.7E–8, 20.33, 0.3084, and 0.099 for Mg%, TS, WR, and IE, respectively.

## 4 Conclusions

In the current study, 3 groups of experiments were conducted to produce CGI with varying Mg contents and CEs. The hardness, TS, wear, and impact strength of the manufactured specimens were mechanically tested. the regression analysis and ANNs model were used to estimate the correlation between the hardness with the wear rate and impact strength of CGI at varying Mg contents and CE values. The following are the primary conclusions:

1. As the Mg content, % in the tested CGI increases, the hardness and impact energy values increase, whereas the wear rate values decrease.
2. The hardness values improve as the CE in the CGI increases; however, the impact strength and wear rate values decrease, and the optimum hardness, impact strength and wear rate values for the produced CGI are obtained at a CE of 4.0.
3. As the CE in the prepared CGI increases from 3.5 to 4.5 %, the hardness increases from 49.22 to 55.86 %, the wear rate from 36.32 to 40.8 % and impact energy values from 42.85 to 65.12 %.
4. The polynomial and linear correlations of the wear rate with hardness are excellent in all experimental outcomes, with an R^2^ of 0.894 and those of the impact strength with hardness are poor with an R^2^ of 0.266.
5. The R^2^ values for the prepared CGI at different CEs are 0.936–0.969 and 0.941–0.962 for the impact strength and wear rate, respectively. An increase at the CE has an effect on hardness in all the cases studied.
6. The neural network algorithm model has a high prediction precision for determining the Mg% content, impact strength and wear rate values of the prepared CGI based on hardness.
7. The calculated MSE for the actual and computed data taken from the used ANNs model at CE = 4 are 3.7E–8, 20.33, 0.099, and 0.3084 for Mg%, TS, Impact strength and wear rate, respectively.
8. Comparisons between the predicted and actual outcomes of hardness, tensile, impact strength and wear rate indicate that good agreement.

## Data availability statement

Data included in article/supplementary material/referenced in article.

## CRediT authorship contribution statement

**Gamal M.A. Mahran:** Funding acquisition, Project administration, Supervision, Writing – review & editing, Conceptualization, Data curation, Methodology, Validation. **Abdel-Nasser Mohamed Omran:** Conceptualization, Data curation, Formal analysis, Methodology, Writing – original draft.

## Declaration of competing interest

The authors declare that they have no known competing financial interests or personal relationships that could have appeared to influence the work reported in this paper.
